# Comparison of Neoatherosclerosis and Neovascularization of Restenosis after Drug-Eluting Stent Implantation: An Optical Coherence Tomography Study

**DOI:** 10.31083/j.rcm2412341

**Published:** 2023-11-30

**Authors:** Chancui Deng, Zhijiang Liu, Wei Zhang, Yi Deng, Hanlin Liu, Zhixun Bai, Jidong Rong, Wenwen Deng, Ning Gu, Youcheng Shen, Xingwei Hu, Yongchao Zhao, Ranzun Zhao, Bei Shi

**Affiliations:** ^1^Department of Cardiology, Affiliated Hospital of Zunyi Medical University, 563000 Zunyi, Guizhou, China

**Keywords:** optical coherence tomography, in-stent restenosis, neoatherosclerosis, neovascularization

## Abstract

**Background:**

Neoatherosclerosis (NA) is associated with stent failure. However, systematic studies on the manifestations of NA and neovascularization (NV) at different stages after drug-eluting stent (DES) implantation are lacking. Moreover, the relationship between NA and NV in in-stent restenosis (ISR) has not been reported. This study aimed to characterize NA and NV in patients with ISR at different post-DES stages and compare the association between NA and NV in ISR lesions.

**Methods:**

A total of 227 patients with 227 lesions who underwent follow-up optical coherence tomography before percutaneous coronary intervention for DES ISR were enrolled and divided into early (E-ISR: <1 year), late (L-ISR: 1–5 years), and very-late (VL-ISR: >5 years) ISR groups. Furthermore, ISR lesions were divided into NV and non-NV groups according to the presence of NV.

**Results:**

The prevalence of NA and NV was 52.9% and 41.0%, respectively. The prevalence of lipidic NA (E-ISR, 32.7%; L-ISR, 50.0%; VL-ISR, 58.5%) and intimal NV (E-ISR, 14.5%; L-ISR, 30.8%; VL-ISR, 38.3%) increased with time after stenting. NA was higher in ISR patients with NV lesions than in those without (*p* < 0.001). Patients with both ISR and NV had a higher incidence of macrophage infiltration, thin-cap fibroatheroma, intimal rupture, and thrombosis (*p* < 0.01).

**Conclusions:**

Progression of lipidic NA was associated with L-ISR and VL-ISR but may not be related to calcified NA. NA was more common in ISR lesions with NV; its formation may substantially promote NA progression and plaque instability.

## 1. Introduction

Despite the ongoing evolution and various iterations of drug-eluting stent (DES) technologies, the prevalence of in-stent restenosis (ISR) remains high, accounting for approximately 10% of percutaneous coronary interventions (PCI) [1, 2, 3]. Therefore, even the latest DES implants cannot prevent stent failure. With the development and wide application of endovascular imaging techniques, increasing evidence shows that in-stent neoatherosclerosis (NA) is a major cause of stent failure, especially in the extended phase after stent implantation [4, 5, 6, 7]. Neovascularization (NV) is associated with plaque vulnerability [8, 9]; however, no reports have been found on the relationship between NA and NV in ISR lesions. Moreover, elevated low-density lipoprotein cholesterol (LDL-C) levels have been reported to increase the risk of plaque rupture in de novo lesions. Lee *et al*. [5] showed LDL-C levels >70 mg/dL (>1.8 mmol/L) to be associated with NA formation. In real clinical practice, the effect of LDL-C level control on optical coherence tomography (OCT) characteristics of ISR lesions remains unclear. Finally, previous studies have reported the relationship between neointima characteristics and stent implantation time [10, 11, 12]; however, the specific manifestations of NA and NV in ISR at different stages and their relationship remain unclear. OCT is the preferred intravascular imaging method for diagnosing NA *in vivo * [13, 14, 15]. Therefore, to understand the mechanism and time course of DES ISR, this study used OCT to systematically investigate the neointimal characteristics of early, late, and very-late ISR, particularly focusing on the specific manifestations of NA and its relationship with NV, which is expected to provide more insights into the mechanism of ISR.

## 2. Materials and Methods

### 2.1 Study Population

**Fig. 1.**
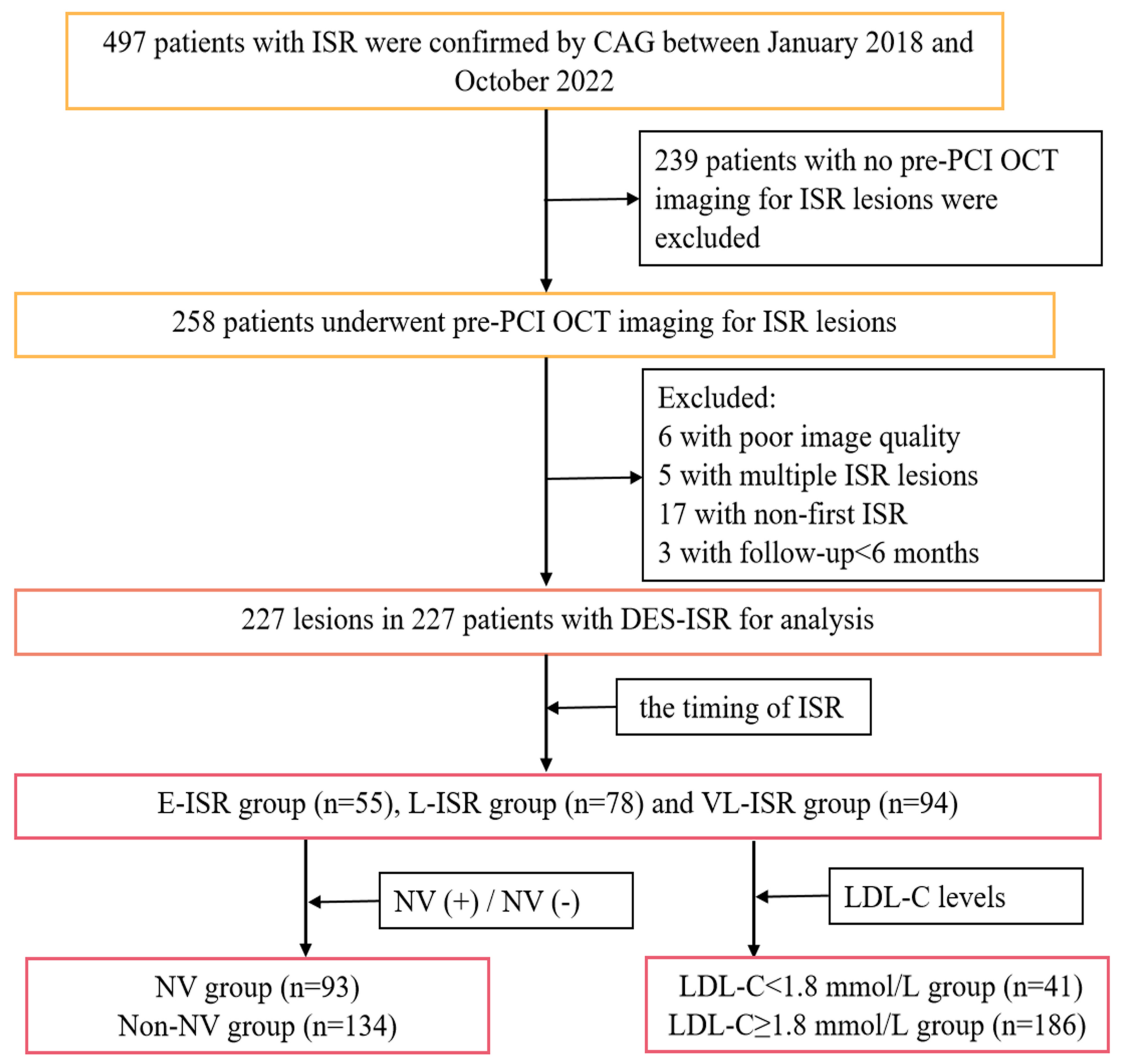
**Study flow diagram**. ISR, in-stent restenosis; CAG, coronary angiography; DES, drug-eluting stents; E-ISR, early in-stent restenosis; LDL-C, low-density lipoprotein cholesterol; L-ISR, late in-stent restenosis; NV, neovascularization; OCT, optical coherence tomography; PCI, percutaneous coronary intervention; VL-ISR, very late in-stent restenosis.

In this single-center retrospective study, we consecutively screened 497 patients with ISR confirmed by coronary angiography (CAG) at the Affiliated Hospital of Zunyi Medical University between January 2018 and October 2022. ISR was defined as a percent diameter stenosis exceeding 50% within the stent implantation segment [10, 11, 12]. The inclusion criteria were first ISR on CAG follow-up and availability of OCT images before re-PCI. The exclusion criteria were combined multiple ISRs, ISR occurring <6 months after stenting, and poor-quality OCT images. Ultimately, 227 patients were included for analysis (Fig. 1), and details are displayed in the **Supplementary Materials**. The **Supplementary Materials** also show definitions of early in-stent restenosis (E-ISR), late in-stent restenosis (L-ISR), and very-late in-stent restenosis (VL-ISR) and reasons for conducting follow-up CAG. The NA and NV characteristics of ISR at different periods and the relationship between NA and NV in ISR lesions were compared. Additionally, to evaluate lipid control on OCT features of ISR lesions at follow-up, patients with ISR were further divided according to LDL-C levels into <1.8 mmol/L and ≥1.8 mmol/L groups [5].

### 2.2 OCT Image Acquisition and Analysis

**Fig. 2.**
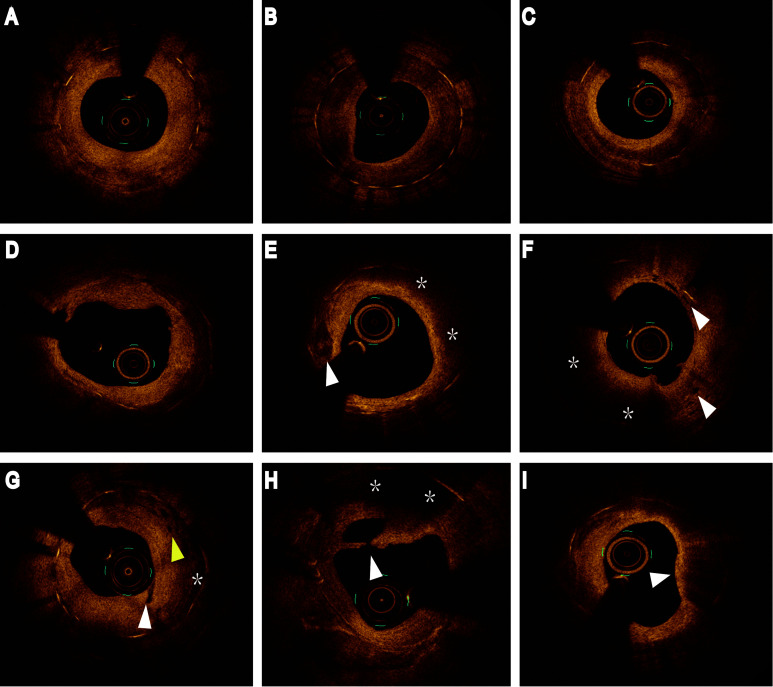
**Representative optical coherence tomography images of restenosis**. (A) Homogeneous neointima. (B) Heterogeneous neointima. (C) Layered neointima. (D) Calcified neoatherosclerosis. (E) Both lipidic (asterisks) and calcified neoatherosclerosis (arrowhead). (F) Neoatherosclerosis (asterisks) with neovascularization (arrowheads). (G) Intraintima neovascularization (white arrowhead), peri-stent neovascularization (yellow arrowhead), and PLIA (asterisk). (H) Lipidic neoatherosclerosis (asterisks) with intimal disruption (arrowhead). (I) Macrophage infiltration (arrowhead). PLIA, peri-stent low intensity area.

Quantitative coronary angiographic (QCA) and OCT analyses are presented in the **Supplementary Materials**. NA was defined as neointimal formation in the presence of lipids or calcifications in ≥3 consecutive frames on OCT images [5, 16]. Lipidic neointima was defined as an intimal area with a diffused border and signal-poor region with marked attenuation [17]. Calcified neointima was defined as a well-defined area with well-defined contours and poor signal intensity [18]. Plaque type was considered mixed if there were both lipidic and calcific NA within the stenosis segment [16]. NV was defined as small (µm) vesicular or tubular structures identified on at least three consecutive cross-sectional OCT images [8, 9, 17, 19]. NV was considered endointimal if located in the superficial 50% of the neointimal thickness or peri-stent if located in the deep 50% of the neointimal thickness; in addition, some of the stenosis segments may have both endointimal and peri-stent NV. Other definitions of neointimal morphologies are displayed in the **Supplementary Materials**. Representative OCT images of ISR are displayed in Fig. 2.

### 2.3 Statistical Analysis

Details of data analyses are presented in the **Supplementary Materials**.

## 3. Results

### 3.1 Baseline and Angiographic Characteristics

A total of 227 patients with 227 lesions were included, with 55, 78, and 94 cases in the E-ISR, L-ISR, and VL-ISR groups, respectively. A comparative analysis of the basic data and QCA analysis of the three groups showed no significant differences among the groups, except for the clinical manifestations during OCT follow-up and the time of stent implantation. The number of patients presenting with acute coronary syndrome (ACS) at the early, late, and very late stages of CAG follow-up was 10 (15.4%), 17 (21.8%), and 42 (44.7%), respectively (*p* < 0.001) (Table 1). There were significant differences in median stent implantation time for patients with E-ISR, L-ISR, and VL-ISR (11, 35, and 84 months, respectively; *p* < 0.001). The QCA data are summarized in **Supplementary Table 1**.

**Table 1.** **Baseline characteristics**.

		Overall (n = 227)	E-ISR (n = 55)	L-ISR (n = 78)	VL-ISR (n = 94)	*p* value	*p* value*		
							① vs. ②	① vs. ③	② vs. ③
General information									
	Age, year	64.00 (56.00–71.00)	62.00 (54.00–70.00)	63.00 (53.00–71.00)	66.00 (58.00–72.00)	0.065			
	Male	175 (77.1)	40 (72.7)	60 (76.9)	75 (79.8)	0.612			
	Smoking	125 (55.1)	34 (61.8)	42 (53.8)	49 (52.1)	0.499			
	Hypertension	138 (60.8)	34 (61.8)	44 (56.4)	60 (63.8)	0.602			
	Diabetes mellitus	70 (30.8)	17 (30.9)	18 (23.1)	35 (37.2)	0.135			
	LDL-C (mmol/L)	2.33 (1.95–2.90)	2.27 (1.82–2.70)	2.29 (1.96–2.76)	2.42 (1.99–3.20)	0.139			
	Creatinine (µmol/L)	84.00 (70.00–100.00)	84.00 (69.00–97.00)	84.00 (72.00–96.00)	84.00 (69.00–105.25)	0.832			
	LVEF (%)	56.00 (44.00–61.00)	55.00 (43.00–60.00)	56.00 (42.00–61.00)	56.00 (51.75–60.00)	0.612			
	Time from implantation (months)	38.00 (13.00–72.00)	11.00 (8.00–11.00)	35.00 (24.00–39.00)	84.00 (63.00–120.00)	<0.001	<0.001	<0.001	<0.001
Clinical presentation									
	ACS at stenting	115 (50.7)	32 (58.2)	38 (48.7)	45 (47.9)	0.437			
	ACS at ISR	69 (29.1)	10 (15.4)	17 (21.8)	42 (44.7)	<0.001	0.329	<0.001	0.002
Medication at follow-up									
	Aspirin	185 (81.5)	48 (87.3)	66 (84.6)	71 (75.5)	0.14			
	P2Y12 inhibitor	174 (76.7)	47 (85.5)	59 (75.6)	68 (72.3)	0.182			
	Statin	195 (85.9)	50 (90.9)	70 (89.7)	75 (79.8)	0.082			

Data are expressed as the median [interquartile range] or n (%). *A *p* value of <0.017 was considered significant. ISR, in-stent restenosis; ACS, acute coronary syndrome; E-ISR, early in-stent restenosis; LDL-C, low-density lipoprotein cholesterol; L-ISR, late in-stent restenosis; LVEF, left ventricular ejection fraction; VL-ISR, very late in-stent restenosis. ①: E-ISR; ②: L-ISR; ③: VL-ISR.

### 3.2 OCT Findings of the Entire Stent and Minimum Lumen Area (MLA) Site

#### 3.2.1 Analysis of the Entire Stent

**Fig. 3.**
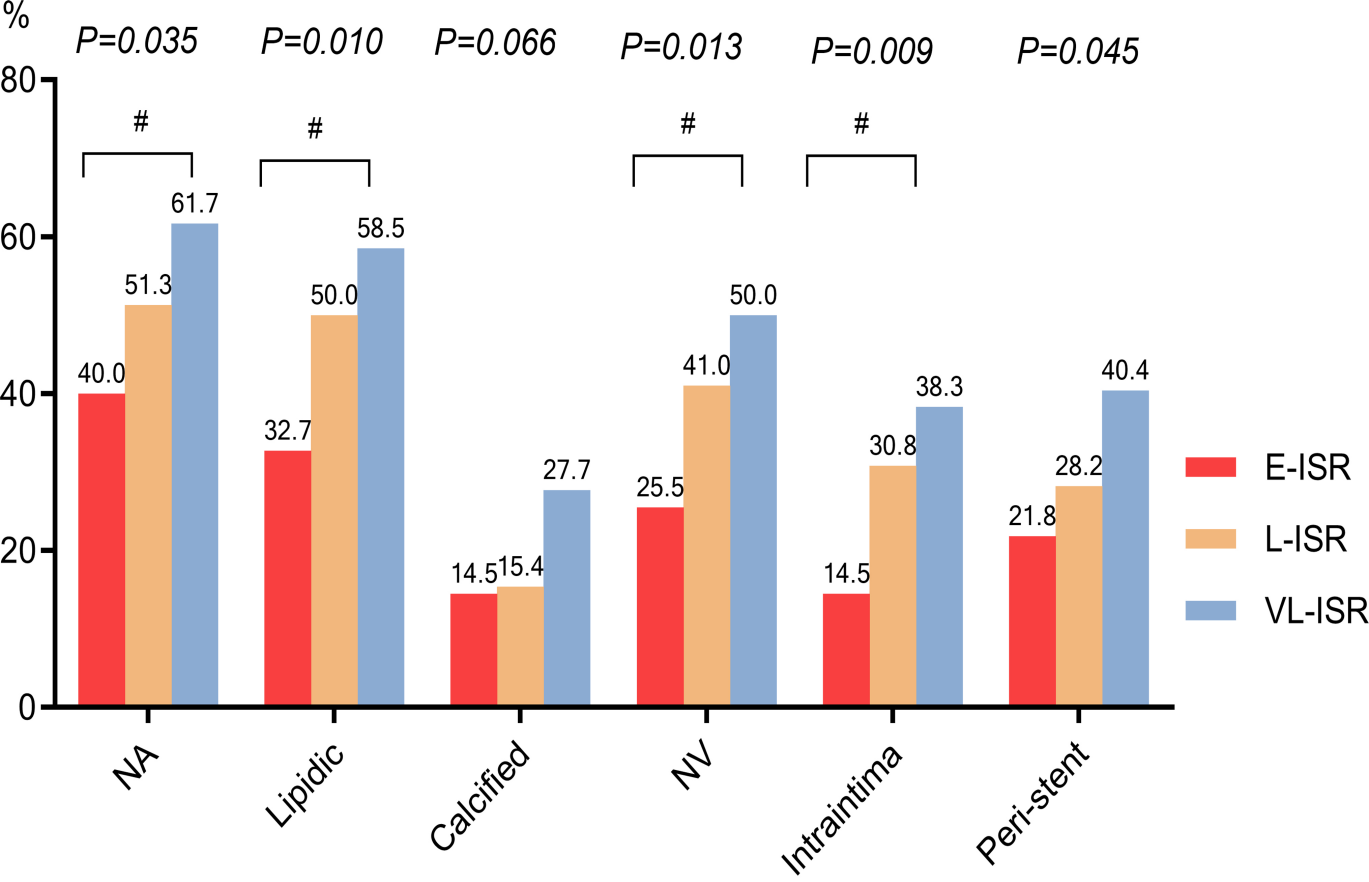
**OCT analysis of the entire stent**. The prevalence of NA and NV in an entire stent. E-ISR, early in-stent restenosis; L-ISR, late in-stent restenosis; NA, neoatherosclerosis; NV, neovascularization; VL-ISR, very late in-stent restenosis; OCT, optical coherence tomography. ^#^*p* < 0.017.

The OCT analysis data for the entire stent are summarized in Table 2 and Fig. 3. The kappa coefficients for inter- and intra-observer agreement for the assessment of NA, lipid NA, calcified NA, NV, intraintima NV, and peri-stent NV were 0.92/0.93, 0.89/0.92, 0.91/0.93, 0.93/0.94, 0.93/0.93, and 0.90/0.93, respectively. No significant differences were observed in the quantitative analysis results among the three groups. In the qualitative analysis, the overall prevalence of NA and NV was 52.9% (49.3% lipidic, 20.3% calcified, and 16.7% were both lipidic and calcific) and 41.0% (intimal, 30.0%; peri-stent, 31.7%; and 20.7% were both intimal and peri-stent), respectively (**Supplementary Fig. 1**). The prevalence of NA (E-ISR, 40.0%; L-ISR, 51.3%; LV-ISR, 61.7%) and NV (E-ISR, 25.5%; L-ISR, 41.0%; VL-ISR 50.0%) increased with time after stenting. NA mainly manifested as lipidic, while the prevalence of calcified NA was not significantly different among the three groups. Moreover, heterogeneous intima, thin-cap fibroatheroma (TCFA), intimal rupture, plaque erosion, macrophage infiltration, red thrombus, and white thrombus were more common in the VL-ISR than in the E-ISR group (*p* < 0.05). Although TCFA, intimal rupture, plaque erosion, macrophage infiltration, red thrombus, and white thrombus showed no significant differences between the E-ISR and L-ISR groups or between the L-ISR and VL-ISR groups, there was an increasing trend with stent time.

**Table 2.** **Analysis of the entire stent by OCT**.

			Overall (n = 227)	E-ISR (n = 55)	L-ISR (n = 78)	VL-ISR (n = 94)	*p* value	*p* value*		
								① vs. ②	① vs. ③	② vs. ③
Quantitative analysis										
	Mean lumen area, ${\mathrm{mm}}^{2}$		3.46 (2.41–4.72)	3.38 (2.38–4.78)	3.52 (2.50–4.70)	3.47 (2.34–4.70)	0.405			
	Mean stent area, ${\mathrm{mm}}^{2}$		7.12 (5.81–8.58)	7.02 (5.60–8.29)	7.21 (6.02–8.58)	7.11 (5.77–8.90)	0.081			
	Neointimal area, ${\mathrm{mm}}^{2}$		3.31 (2.33–4.72)	3.23 (2.34–4.66)	3.36 (2.39–4.73)	3.32 (2.27–4.72)	0.688			
	Neointimal burden (%)		49.13 (36.06–62.88)	48.40 (36.32–61.99)	49.41 (37.20–61.88)	49.28 (35.25–64.43)	0.903			
Qualitative analysis										
	Predominantly homogeneous		105 (46.3)	41 (74.5)	38 (48.7)	26 (27.7)	<0.001	0.03	<0.001	0.004
	Predominantly		122 (53.7)	14 (25.5)	40 (51.3)	68 (72.3)	<0.001	0.03	<0.001	0.004
	heterogeneous									
	Layered		45 (19.8)	9 (16.4)	11 (14.1)	25 (26.6)	0.094			
	NA		120 (52.9)	22 (40.0)	40 (51.3)	58 (61.7)	0.035	0.199	0.01	0.169
		Lipidic	112 (49.3)	18 (32.7)	39 (50.0)	55 (58.5)	0.01	0.047	0.002	0.264
		Calcified	46 (20.3)	8 (14.5)	12 (15.4)	26 (27.7)	0.066			
	TCFA		45 (19.8)	5 (9.1)	13 (16.7)	27 (28.7)	0.01	0.208	0.005	0.062
	Intimal disruption		41 (18.1)	4 (7.3)	12 (15.4)	25 (26.6)	0.009	0.157	0.004	0.075
	Plaque erosion		63 (27.8)	8 (14.5)	22 (28.2)	33 (35.1)	0.026	0.063	0.007	0.334
	Macrophage		43 (18.9)	3 (5.5)	13 (16.7)	27 (28.7)	0.002	0.05	0.001	0.062
	Cholesterol crystal		35 (15.4)	5 (9.1)	12 (15.4)	18 (19.1)	0.26			
	PLIA		31 (13.7)	10 (18.2)	12 (15.4)	9 (9.6)	0.289			
	NV		93 (41.0)	14 (25.5)	32 (41.0)	47 (50.0)	0.013	0.063	0.003	0.24
		Intraintima	68 (30.0)	8 (14.5)	24 (30.8)	36 (38.3)	0.009	0.031	0.002	0.302
		Peri-stent	72 (31.7)	12 (21.8)	22 (28.2)	38 (40.4)	0.045	0.406	0.02	0.094
	Thrombus		58 (25.6)	6 (10.9)	17 (21.8)	35 (37.2)	0.001	0.102	0.001	0.028
		Red	39 (17.2)	3 (5.5)	12 (15.4)	24 (25.5)	0.006	0.075	0.002	0.103
		White	49 (21.6)	6 (10.9)	15 (19.2)	28 (29.8)	0.021	0.195	0.008	0.111

Data are expressed as the median [interquartile range] or n (%). *A *p* value of <0.017 was considered statistically significant. E-ISR, early in-stent restenosis; L-ISR, late in-stent restenosis; NA, neoatherosclerosis; NV, neovascularization; OCT, optical coherence tomography; PLIA, peri-low intensity area; TCFA, thin-cap fibroatheroma; VL-ISR, very late in-stent restenosis. ①: E-ISR; ②: L-ISR; ③: VL-ISR.

#### 3.2.2 Analysis of MLA Site

The OCT analysis data for the MLA site are summarized in** Supplementary Table 2**. The results are similar to those of the entire stent analysis. Quantitative analysis showed no significant differences among the three groups. Qualitative analysis showed that from E-ISR to VL-ISR, the incidences of NA and NV increased gradually, NA was still mainly lipidic, and NV mostly manifested as endointimal microvessels. Moreover, heterogeneous intima, TCFA, intimal rupture, macrophage infiltration, endointimal NV, and red thrombosis were more common in the VL-ISR group.

### 3.3 Relationship between NA and NV

Before comparing the relationship between NA and NV, we first compared the characteristics of NA and non-NA patients, and found that the NA group had higher LDL-C and creatinine levels and longer stent implantation time compared with those in the non-NA group (*p* < 0.05). No significant differences were observed in diabetes mellitus, stent type, lesion characteristics of CAG, and OCT quantitative analysis of the MLA site between the two groups (*p* > 0.05) (**Supplementary Table 3**).

**Fig. 4.**
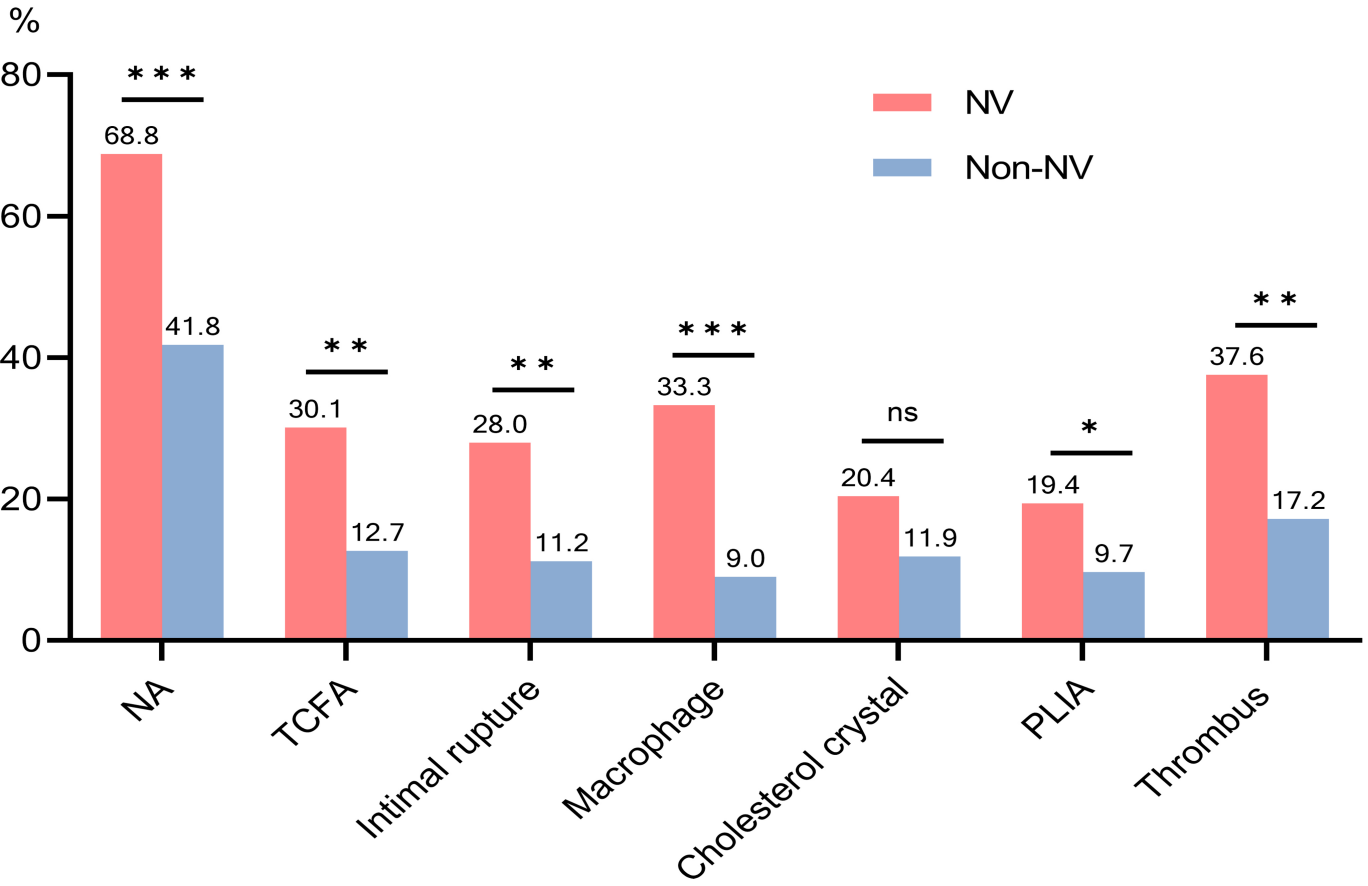
**Comparison of OCT characteristics of ISR lesions in the NV and non-NV groups**. NA, neoatherosclerosis; NV, neovascularization; PLIA, peri-low intensity area; TCFA, thin-cap fibroatheroma; OCT, optical coherence tomography. ***p < 0.001; **p < 0.01; *p < 0.05; ns, not significant.

Next, we compared the relationship between NA and NV and found no significant differences in general clinical data, CAG, and quantitative OCT findings of the MLA site between NV and non-NV groups, except for stent implantation time, MLA, and stent area; the median stent implantation time was longer in the NV group than in the non-NV group (60.0 months vs. 36.0 months, *p* = 0.008); the MLA and stent area of the NV group were larger than those of the non-NV group (*p* < 0.05). Qualitative OCT analysis showed that the prevalence of NA was higher in ISR patients with NV lesions than in those without (68.8% vs. 41.8%, *p* < 0.001) (Fig. 4). Moreover, patients with ISR combined with NV had a higher incidence of heterogeneous intima, macrophage infiltration, TCFA, intimal rupture, peri-stent low intensity area (PLIA), and thrombosis (*p* < 0.05) (Table 3, Fig. 4).

**Table 3.** **Plaque features evaluation based on ISR lesions with or without NV**.

			Overall (n =227)	NV (n = 93)	Non-NV (n = 134)	*p* value
General information						
	Age, year		64.00 (56.00–71.00)	64.00 (57.00–71.50)	63.00 (54.00–70.25)	0.233
	Male		175 (77.1)	72 (77.4)	103 (76.9)	0.922
	Smoking		125 (55.1)	50 (53.8)	75 (56.0)	0.742
	Hypertension		138 (60.8)	57 (61.3)	81 (60.4)	0.898
	Diabetes mellitus		70 (30.8)	32 (34.4)	38 (28.4)	0.332
	LDL-C (mmol/L)		2.33 (1.95–2.90)	2.27 (1.83–3.05)	2.38 (1.98–2.88)	0.389
	Creatinine (µmol/L)		84.00 (70.00–100.00)	85.00 (71.50–100.00)	83.50 (69.00–100.25)	0.42
	LVEF (%)		56.00 (44.00–61.00)	56.00 (50.00–61.00)	56.00 (42.75–60.00)	0.136
	Time from implantation (months)		38.00 (13.00–72.00)	60.00 (24.00–96.00)	36.00 (11.00–63.25)	0.008
CAG finding						
	Length, mm		12.10 (8.70–17.90)	11.80 (8.75–17.10)	12.35 (8.60–18.53)	0.564
	Reference vessel diameter, mm		3.18 ± 0.41	3.23 ± 0.42	3.14 ± 0.39	0.107
	MLD, mm		1.14 ± 0.22	1.15 ± 0.21	1.13 ± 0.22	0.496
	Diameter stenosis (%)		63.48 (60.06–67.68)	63.31 (59.99–67.63)	63.56 (60.05–67.70)	0.962
	Previous stent type					0.416
		First-generation DES	172 (75.8)	72 (77.4)	100 (74.6)	
		New-generation DES	44 (19.4)	15 (16.1)	29 (21.6)	
		Unknown	11 (4.8)	6 (6.5)	5 (3.7)	
OCT finding						
	MLA, ${\mathrm{mm}}^{2}$		1.64 ± 0.55	1.75 ± 0.58	1.57 ± 0.52	0.016
	Stent area (MLA site), ${\mathrm{mm}}^{2}$		6.74 ± 1.97	7.11 ± 2.13	6.47 ± 1.81	0.016
	Neointimal area (MLA site), ${\mathrm{mm}}^{2}$		4.80 (3.84–6.12)	5.12 (4.05–6.34)	4.66 (3.80–5.93)	0.111
	Neointimal burden (MLA site), %		74.54 ± 8.79	74.34 ± 8.51	74.68 ± 9.00	0.773
	Predominantly homogeneous		105 (46.3)	31 (33.3)	74 (55.2)	0.001
	Predominantly heterogeneous		122 (53.7)	62 (66.7)	60 (44.8)	
	NA		120 (52.9)	64 (68.8)	56 (41.8)	<0.001
	Non-NA		107 (47.1)	29 (31.2)	78 (58.2)	<0.001
	TCFA		45 (19.8)	28 (30.1)	17 (12.7)	0.001
	Intimal rupture		41 (18.1)	26 (28.0)	15 (11.2)	0.001
	Macrophage		43 (18.9)	31 (33.3)	12 (9.0)	<0.001
	Cholesterol crystal		35 (15.4)	19 (20.4)	16 (11.9)	0.082
	PLIA		31 (13.7)	18 (19.4)	13 (9.7)	0.037
	Thrombus		58 (25.6)	35 (37.6)	23 (17.2)	0.001

Data are expressed as the median [interquartile range], mean ± SD (standard deviation), or n (%). CAG, coronary angiography; DES, drug-eluting stent; ISR, in-stent restenosis; LVEF, left ventricular ejection fraction; MLA, minimum lumen area; MLD, minimal lumen diameter; LDL-C, low-density lipoprotein cholesterol; NA, neoatherosclerosis; NV, neovascularization; OCT, optical coherence tomography; PLIA, peri-low intensity area; TCFA, thin-cap fibroatheroma.

### 3.4 Clinical Data, CAG, and OCT Findings Concerning LDL-C Levels

**Fig. 5.**
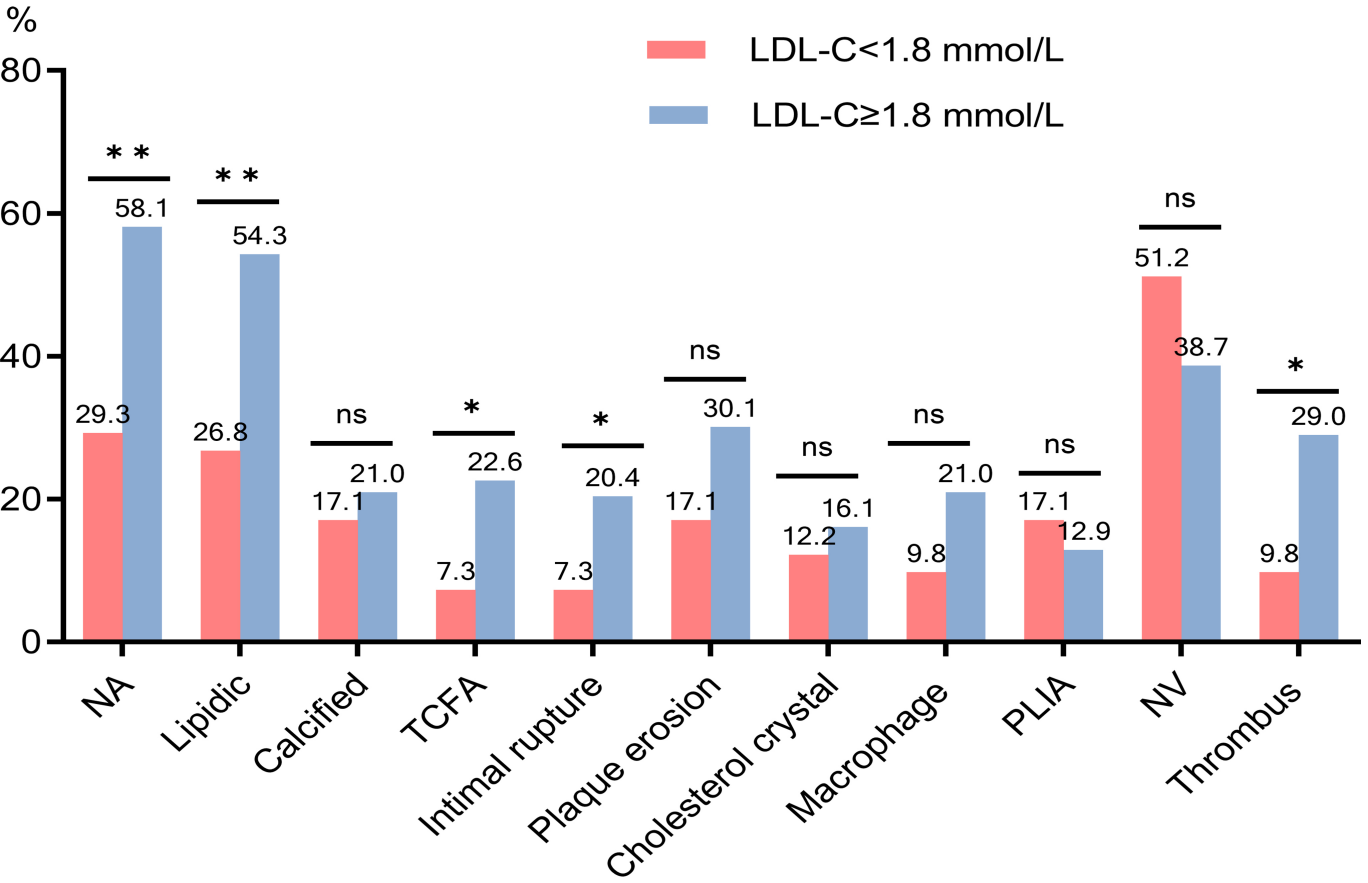
**Comparison of plaque characteristics between LDL-C <1.8 mmol/L and LDL-C ≥1.8 mmol/L groups**. LDL-C, low-density lipoprotein cholesterol; NA, neoatherosclerosis; NV, neovascularization; PLIA, peri-low intensity area; TCFA, thin-cap fibroatheroma. **p < 0.01; *p < 0.05; ns, not significant.

Comparison of the general data and CAG findings between the two groups revealed that the prevalence of diabetes mellitus (DM) was higher in the LDL-C <1.8 mmol/L group. The OCT data of the two groups showed that the incidences of lipidic NA, heterogeneous intima, TCFA, intimal rupture, and thrombus were higher in the LDL-C ≥1.8 mmol/L group (*p* < 0.05). No significant differences were observed between the two groups in the prevalence of calcified NA, plaque erosion, macrophage infiltration, NV, cholesterol crystal, and PLIA (*p* > 0.05) (Table 4, Fig. 5).

**Table 4.** **Comparison of clinical data, CAG, and OCT findings in patients with ISR concerning LDL-C levels**.

			Overall (n = 227)	LDL-C <1.8 mmol/L (n = 41)	LDL-C ≥1.8 mmol/L (n = 186)	*p* value
General information						
	Age, year		64.00 (56.00–71.00)	65.00 (56.00–73.00)	64.00 (55.75–71.00)	0.488
	Male		175 (77.1)	35 (85.4)	140 (75.3)	0.164
	Smoking		125 (55.1)	25 (61.0)	100 (53.8)	0.401
	Hypertension		138 (60.8)	24 (58.5)	114 (61.3)	0.744
	Diabetes mellitus		70 (30.8)	18 (43.9)	52 (28.0)	0.045
	Creatinine (µmol/L)		84.00 (70.00–100.00)	85.00 (72.00–100.50)	84.00 (69.00–100.00)	0.471
	LVEF (%)		56.00 (44.00–61.00)	58.00 (43.00–62.50)	55.00 (44.75–60.00)	0.306
	Time from implantation (months)		38.00 (13.00–72.00)	36.00 (11.00–68.50)	48.00 (15.00–84.00)	0.149
	Previous stent type					0.406
		First-generation DES	172 (75.8)	28 (68.3)	144 (77.4)	
		New-generation DES	44 (19.4)	11 (26.8)	33 (17.7)	
		Unknown	11 (4.8)	2 (4.9)	9 (4.8)	
CAG finding						
	Length, mm		12.10 (8.70–17.90)	10.40 (8.45–16.20)	12.85 (8.80–18.33)	0.058
	Reference vessel diameter, mm		3.18 ± 0.41	3.23 ± 0.42	3.17 ± 0.40	0.341
	MLD, mm		1.14 ± 0.22	1.16 ± 0.23	1.13 ± 0.21	0.515
	Diameter stenosis (%)		63.48 (60.06–67.68)	61.95 (59.33–68.09)	63.64 (60.24–67.55)	0.402
OCT finding						
	Predominantly homogeneous		105 (46.3)	34 (82.9)	71 (38.2)	<0.001
	Predominantly heterogeneous		122 (53.7)	7 (17.1)	115 (61.8)	<0.001
	NA		120 (52.9)	12 (29.3)	108 (58.1)	0.001
		Lipidic	112 (49.3)	11 (26.8)	101 (54.3)	0.001
		Calcified	46 (20.3)	7 (17.1)	39 (21.0)	0.574
	TCFA		45 (19.8)	3 (7.3)	42 (22.6)	0.026
	Intimal rupture		41 (18.1)	3 (7.3)	38 (20.4)	0.048
	Plaque erosion		63 (27.8)	7 (17.1)	56 (30.1)	0.092
	Macrophage		43 (18.9)	4 (9.8)	39 (21.0)	0.097
	Cholesterol crystal		35 (15.4)	5 (12.2)	30 (16.1)	0.528
	PLIA		31 (13.7)	7 (17.1)	24 (12.9)	0.482
	NV		93 (41.0)	21 (51.2)	72 (38.7)	0.14
		Intraintima	68 (30.0)	11 (26.8)	57 (30.6)	0.629
		Peri-stent	72 (31.7)	18 (43.9)	54 (29.0)	0.064
	Thrombus		58 (25.6)	4 (9.8)	54 (29.0)	0.01
		Red	39 (17.2)	2 (4.9)	37 (19.9)	0.021
		White	49 (21.6)	4 (9.8)	45 (24.2)	0.042

Data are expressed as the median [interquartile range], mean ± SD (standard deviation), or n (%). ISR, in-stent restenosis; CAG, coronary angiography; DES, drug-eluting stent; LDL-C, low-density lipoprotein cholesterol; LVEF, left ventricular ejection fraction; MLD, minimal lumen diameter; NA, neoatherosclerosis; NV, neovascularization; OCT, optical coherence tomography; PLIA, peri-low intensity area; TCFA, thin-cap fibroatheroma.

## 4. Discussion

The main findings of the study are as follows: (1) the prevalence of lipidic (not calcified) NA and intimal NV increased over time after stenting. Additionally, the homogeneous intima decreased gradually, while the heterogeneous intima increased from E-ISR to VL-ISR. TCFA, intimal rupture, plaque erosion, macrophage infiltration, and thrombus were more common in the VL-ISR group than in the E-ISR group; (2) the prevalence of NA was higher in patients with ISR and NV lesions than in those without. Moreover, patients with ISR plus NV had a higher incidence of macrophage infiltration, TCFA, intimal rupture, and thrombosis; (3) patients with ISR with poorly controlled LDL-C levels had a higher incidence of plaque vulnerability than those with LDL-C <1.8 mmol/L.

### 4.1 Prevalence of NA and Temporal Patterns

A previous study found that the incidences of NA after the first- and second-generation of DES implants were 45.5% and 10.8%, respectively; moreover, the incidence of NA gradually increased with the extension of follow-up time [5]. Nakamura *et al*. [4] reported the NA incidence of 47.0% among 64 bare-metal stent (BMS) ISR and 241 DES ISR lesions. Chen *et al*. [16] reported an NA incidence as high as 75% after >7 years of stent implantation. Therefore, emerging evidence suggests that the incidence of NA increases with stent implantation time. In this study, the overall prevalence of NA was 52.9%, and it exhibited a time-dependent pattern: E-ISR, 40.0%; L-ISR, 51.3%; VL-ISR, 61.7%. The incidence of NA and its time dependence are similar to those previously reported.

Although evidence suggests that the incidence of NA is time-dependent, systematic studies on the manifestations of NA at different stages are lacking. Previous studies by Yonetsu *et al*. [20] reported that the incidence of lipid-rich neointima (lipid NA) in BMS/DES was time-dependent, with 8%/37%, 28%/63%, and 77%/75% in the early (<9 months), middle (9–48 months), and delayed (>48 months) stages, respectively. Jinnouchi *et al*. [12] also reported that after second-generation DES implantation, the frequency of lipid-laden neointima was significantly higher in the L-ISR (beyond one year) than in the E-ISR group (within one year). In this study, the time dependence of NA was also observed, specifically manifested as lipid NA. Moreover, Garcia-Guimaraes *et al*. [21] demonstrated that calcified NA was predominantly observed in very late ISR cases (>3 years). In contrast, our study data suggested a trend of increasing calcified NA from early ISR to very late ISR; however, this observation did not reach statistical significance. Another study found that the presence of ISR with calcified nodule formation (i.e., calcified NA) was associated with female sex, combined hemodialysis, calcified lesions, and stent malapposition, but not with stent implantation time [22]. Our results are inconsistent with those of Garcia-Guimaraes *et al*. [21], which may be related to the use of a retrospective design with a small sample size in the present study or the phenomenon that calcified NA is not associated with stent implantation time but is related to whether the *in situ* lesions are calcified. Therefore, our data preliminarily revealed that the progression of lipid NA is primarily associated with L-ISR and VL-ISR but may not be associated with calcified NA, which needs to be confirmed in large-sample randomized prospective trials.

### 4.2 NA, Stent Failure, and Major Adverse Cardiovascular Events (MACEs)

Previous studies showed that first-generation DES implants significantly reduced the prevalence of BMS-associated ISR but also increased the incidence of stent thrombosis [23]. Second-generation DES is associated with fewer stent thrombotic events, but NA formation remained unavoidable [24, 25]. Lee *et al*. [5] also showed that second-generation DES did not prevent NA better than the first-generation DES. Furthermore, the development of NA after DES implantation has been described as a late catch-up phenomenon, as it has been observed that neointimal growth is highly inhibited in the first year after DES implantation; however, subsequently, it shows sustained progression, accompanied by rapid lipid-laden macrophage deposition, thus becoming the final common pathway for stent failure in the late stages [6, 26]. Therefore, both pathological studies and endovascular imaging findings have shown that NA formation was an important cause of late failure of BMS, first- and second-generation DES [6, 27]. In an OCT analysis of 2139 patients with ACS, Amabile *et al*. [28] showed that NA is common in patients with very late stent thrombosis. Habara *et al*. [10] showed that VL-ISR (>5 years) had a significantly higher incidence of heterogeneous intima, NV, intimal rupture, and red thrombus than E-ISR (<1 year). Furthermore, in their first-generation DES ISR study, Habara *et al*. [11] showed a gradual increase in TCFA, intimal rupture, and intimal NV from early to late and very late post-operative periods. Jinnouchi *et al*. [12] reported that after second-generation DES implantation, the frequency of neointima with lipid-laden tissue, macrophage infiltration, NV, and TCFA were significantly higher in the L-ISR than in the E-ISR group. This study also showed that heterogeneous intima, TCFA, intimal rupture, plaque erosion, macrophage infiltration, and thrombus were more common in the VL-ISR group than in the E-ISR group. Similarly, the results of the MLA site analysis were similar to those of the entire stent analysis.

Consequently, NA formation may lead to stent failure and can trigger MACEs [29, 30, 31]. In this study, the number of patients presenting with ACS at follow-up was significantly higher in the VL-ISR group than in the E-ISR group; this may be related to the significantly higher prevalence of NA in the VL-ISR than in the E-ISR group. The incidence of NA with intimal rupture and thrombus in the VL-ISR group (21 intimal ruptures and 30 thrombi among 58 NA lesions) was significantly higher than that in the E-ISR group (3 intimal ruptures and 5 thrombi among 22 NA lesions), suggesting that NA plays an important role in stent failure and MACE. Notably, both the incidence of NA and NV are time-dependent, and there may be a potential relationship between them, but no reports have been found on the relationship between NA and NV in ISR lesions. Based on an *in situ* lesion study, the incidence of NA is reportedly associated with the formation of NV [32]. Therefore, we hypothesized that there might be a correlation between the occurrence of NA and the presence of NV in ISR lesions. To investigate and verify this hypothesis, we further explored the relationship between NA and NV in ISR lesions.

### 4.3 Relationship between NA and NV

NV has been regarded as an important pathway for the delivery of erythrocytes and inflammatory cells involved in lipid plaque formation and has been identified as a contributor of plaque vulnerability [33]. In this study, NV, especially intraintimal NV, increased gradually from E-ISR to VL-ISR. Another study found that NV dilatation in the plaques for native coronary arteries was closely related to plaque vulnerability; plaques with NV have thinner fibrous caps and a higher incidence of plaque rupture [8]. NV inhibition effectively prevents atherosclerosis *in situ * [34]. Lipid-laden intima is thought to be closely associated with intimal NV after BMS implantation [17]. Tian *et al*. [32] also reported that NA was more common in stents with NV within the intima. In addition, Gao *et al*. [35] reported no difference in the incidence of NA between diabetic and non-diabetic patients, but NV in NA lesions was more common in diabetic patients. Our results also showed that the prevalence of NA was similar in diabetic and non-diabetic patients, and no difference in the incidence of NA was observed in diabetic patients with and without NV lesions; this may be related to the inclusion of a special population (ISR patients) in this study, which is expected to be further confirmed by randomized studies. Significantly, we found that NA was more frequently observed in ISR patients with NV lesions. Moreover, patients with ISR plus NV had higher incidences of macrophage infiltration, TCFA, intimal rupture, and thrombosis. These findings suggest that NV formation and dilatation in ISR lesions may be associated with NA progression and plaque vulnerability.

### 4.4 Effect of LDL-C Level on OCT Characteristics of ISR Lesions

LDL-C is well known to be involved in atherosclerosis development and progression, and high levels promote cardiovascular events. *In situ* studies have shown that active control of LDL-C levels can stabilize or reverse coronary plaque, thus reducing the risk of MACE [36, 37, 38]. A first- and second-generation DES study showed that LDL-C >70 mg/dL was related to NA. Higher LDL-C levels were more prevalent in patients with NA [5], and Nakano *et al*. [39] showed that increased LDL-C levels are associated with plaque rupture in patients with ACS; however, in real clinical practice, the effect of LDL-C level control on OCT characteristics of ISR lesions remains unclear. Therefore, we further compared clinical data and OCT characteristics of different LDL-C levels, and unexpectedly, compared with the LDL-C ≥70 mg/dL (1.8 mmol/L) group, the prevalence of DM was higher in the LDL-C <70 mg/dL group. This may be because ISR patients with DM pay more attention to lipid management. However, when comparing OCT characteristics, ISR patients with poorly controlled LDL-C ≥70 mg/dL had a higher incidence of lipidic NA, heterogeneous intima, TCFA, intimal rupture, and thrombus compared to patients with LDL-C <70 mg/dL. These findings suggest the importance of enhanced lipid management for both *in situ* and ISR lesions. However, large-sample randomized controlled trials are needed to determine target LDL-C levels for different populations in complex clinical settings.

### 4.5 Study Limitations

The current research has some limitations. First, this was a single-center, retrospective study with a relatively limited sample size. Second, some individuals diagnosed with ISR were excluded from the study owing to the absence of OCT imaging, which may introduce a potential selection bias, and the data derived from this study may not represent the wider patient population. Consequently, the findings of this investigation are solely descriptive and intended to generate hypotheses, which require further validation through large-sample randomized prospective trials. Third, not reporting the clinical results of these patients is an important limitation of this study. However, we are currently collecting the clinical follow-up data of these patients, and the data are not yet complete. Therefore, they were not included in the analysis of this study. We will continue to collect and analyze the clinical outcomes of these patients and report the results in future studies. Last, there was a lack of histological validation of neointimal tissue characteristics. Although OCT is the preferred intravascular imaging method for diagnosing NA *in vivo*, it has its own limitations and may not accurately assess qualitative neointimal characteristics.

## 5. Conclusions

Progression of lipidic NA was associated with L-ISR and VL-ISR but may not be related to calcified NA. NV formation may be associated with NA progression and plaque vulnerability in ISR lesions. Moreover, patients with poorly controlled LDL-C had lesions with more vulnerable features; consequently, patients with ISR also need aggressive lipid-lowering therapy. However, further randomized controlled trials are needed to determine target LDL-C levels for different populations in clinical practice.

## Data Availability

The datasets generated during the current study are not publicly available due to their confidential nature. However, they can be obtained from the corresponding author upon reasonable request.

## Abbreviations

ACS, acute coronary syndrome; BMS, bare-metal stent; CAG, coronary angiography; DES, drug-eluting stent; DM, diabetes mellitus; ISR, in-stent restenosis; LAD, left anterior descending artery; LCX, left circumflex artery; LDL-C, low-density lipoprotein cholesterol; LVEF, left ventricular ejection fraction; MACE, major adverse cardiovascular event; MLA, minimum lumen area; MLD, minimal lumen diameter; NA, neoatherosclerosis; NV, neovascularization; OCT, optical coherence tomography; PCI, percutaneous coronary intervention; PLIA, peri-stent low intensity area; QCA, quantitative coronary analysis; RCA, right coronary artery.

## Supplementary Material

Supplementary material associated with this article can be found, in the online version, at https://doi.org/10.31083/j.rcm2412341.
