# Supplementary material for: Comparison of Neoatherosclerosis and Neovascularization of Restenosis after Drug-Eluting Stent Implantation: An Optical Coherence Tomography Study
Source: Rev Cardiovasc Med. 2023 Nov 30;24(12):341. doi: 10.31083/j.rcm2412341 (PMC11272882; doi:10.31083/j.rcm2412341)
Supplement: Supplementary file 1 [file 2153-8174-24-12-341-s1.zip › 2153-8174-24-12-341-s1.docx]

**Supplementary material**

1. **Exclusion of patients**

A total of 239 patients among the 497 individuals diagnosed with in-stent restenosis (ISR) were excluded from the study owing to the absence of optical coherence tomography (OCT) imaging prior to the interventional procedure. The decision to forego OCT imaging was primarily due to financial constraints faced by the majority of these patients with ISR, while a minority faced challenges related to vascular tortuosity, the location of ISR lesions distal to the vessel, complete occlusive lesion, and comorbid conditions such as diabetes and renal failure (creatinine clearance <30 ml/min). In addition, six patients had poor OCT image quality, five patients had multiple ISR lesions (at least two ISR lesions in the same patient), and 17 patients had non-first ISR (16 with second and 1 with third occurrences of ISR at the same lesion site); these patients were excluded as well.

1. **Definition of E-ISR, L-ISR, VL-ISR, and the reasons for performing follow-up coronary angiography**
2. ISR was defined as the first ISR confirmed by coronary angiography (CAG) within one year after stent implantation. L-ISR was defined as the first ISR confirmed by CAG between 1 and 5 years after stent implantation, and no ISR was found in the CAG followed up within one year. VL-ISR was defined as the first ISR confirmed by CAG >5 years after stent implantation, and no ISR was indicated by the previous CAG [[1](#Reference1)]. There are two distinct scenarios that warrant follow-up CAG in the present study. A short-term CAG follow-up (stent implantation time < 1 year) should be conducted as a scheduled postoperative evaluation after stent implantation or evidence of myocardial ischemia. In addition, a long-term CAG follow-up (stent implantation time > 1 year) should be performed as part of routine surveillance after treatment of other lesions or evidence of myocardial ischemia [[1](#Reference1), [2](#Reference2)].

**3. Quantitative coronary angiographic** **analysis**

Lesion length, reference vessel diameter, minimal lumen diameter (MLD), and diameter stenosis were analyzed using quantitative coronary analysis (QCA) software (Artis VC21C, Siemens AG, Berlin, and Munich). The angiographic pattern of ISR was classified according to Mehran’s classification [[3](#Reference3)]. The QCA analysis was performed by two experienced coronary intervention professionals who were blinded to the clinical data of the patients.

**4. OCT image acquisition and analysis**

OCT images of all ISR lesions were obtained using the frequency-domain OCT C7XR/ILUMIEN/ILUMIEN OPTIS intracavitary imaging system (St. Jude Medical, St. Paul, MN, USA). All images in this study were analyzed offline by LightLab OCT imaging Inc., Westford, MA, USA (Light Lab imaging Inc., Westford, MA, USA). All cross-sectional images were preliminarily assessed qualitatively and excluded from the analysis if any part of the stent was outside the field of view, side branches were present, or image quality was poor due to incomplete blood clearance or artifacts. Qualitative analysis was performed for each frame, while quantitative analysis was performed every 1 mm along the entire stent segment. All OCT analysis reports were reviewed by two technicians blinded to patients’ CAG and clinical data. If there were disagreements between the two technicians, a third analyst read the images independently and reached a consensus.

**5. Definition of neointima morphology by optical coherence tomography (OCT)**

The neointima was defined as the tissue between the vascular lumen's margin and the stent trabeculae's inner edge. The neointimal area was calculated as the difference between the stent and lumen areas. The neointimal burden was calculated as [neointimal area ×100] ÷ stent area. A homogenous neointima was defined as an area within the intima with a uniform signal-rich band without focal variation or attenuation. A heterogeneous neointima was defined as the area in the intima with focally changing optical properties and various backscattering patterns.

Plaque rupture was defined as the discontinuity of the fibrous cap of a lipid plaque, accompanied by the formation of a cavity. A red thrombus was defined as a tissue image with a weak signal and high backscattering with shadows. A white thrombus was defined as a tissue image with a strong signal and low backscattering without shadows. TCFA was described as lipid-rich neointima with a fibrous cap thickness at the thinnest part <65 μm and lipid arc >180°. Plaque erosion was defined as an intact fibrous cap without plaque rupture accompanied by thrombosis and an identifiable plaque structure under the thrombosis or an intact fibrous cap, absence of thrombosis in the culprit lesion, irregular luminal surface or presence of thrombosis at the lesion, indistinct plaque structure at the thrombus, and absence of superficial lipids or calcification proximal or distal to the thrombus [[4](#Reference4)]. Macrophages were characterized as having specular or banded structures with high reflection, and strong attenuation on OCT images and radiate shadows often formed behind the specular region with a high signal [[5](#Reference5)]. Peri-low intensity area (PLIA) was defined as the area around the stent strut with a uniformly lower intensity appearance than the surrounding tissue on OCT images without significant signal attenuation behind this area [[6](#Reference6)]. Cholesterol crystals were defined as expanded cholesterol that crystallized when passing from a liquid to a solid state [[7](#Reference7)]. Layered intima was defined as intima with one or more layers of different optical densities, clearly demarcated from the underlying components [[8](#Reference8)].

**6. Statistical analysis**

Data analysis in this study was performed using SPSS (version 20.0, IBM, Armonk, NY). Categorical data are presented as numbers (percentages), and differences between groups were compared using the chi-square test or Fisher’s exact test. The Kolmogorov–Smirnov test was used to evaluate the distribution of continuous data. Normally distributed continuous data are presented as mean ± standard deviation (SD), and the independent sample t-test and one-way analysis of variance (ANOVA) were used to compare differences between two and three groups, respectively. Skewed continuous data are presented as median (interquartile range), and the Mann–Whitney U and Kruskal–Wallis tests were used to compare the differences between two and three groups, respectively. Overall comparisons were considered statistically significant using a 2-sided *p* < 0.05. When the comparisons between the three groups were significant, two-group post-hoc comparisons were performed using Mann–Whitney U test, chi-square test, or Fisher’s exact test, while Bonferroni correction was used to control type I error, and a *p* < 0.017 was considered significant. Furthermore, the inter- and intra-observer reproducibility of the imaging analysis was evaluated using the kappa coefficient.

**References**

1. Habara M, Terashima M, Nasu K, Kaneda H, Inoue K, Ito T, et al. Difference of tissue characteristics between early and very late restenosis lesions after bare-metal stent implantation: an optical coherence tomography study. Circulation Cardiovascular interventions. 2011; 4: 232-8. https://doi.org/10.1161/CIRCINTERVENTIONS.110.959999.
2. Habara M, Terashima M, Nasu K, Kaneda H, Yokota D, Ito T, et al. Morphological differences of tissue characteristics between early, late, and very late restenosis lesions after first generation drug-eluting stent implantation: an optical coherence tomography study. European Heart Journal - Cardiovascular Imaging. 2013; 14: 276-84. https://doi.org/10.1093/ehjci/jes183.
3. Mehran R. Patterns of in-stent restenosis : Angiographic classification and implications for long-term clinical outcome. Circulation. 1999; 100: 1872-8.

<https://doi.org/> 10.1161/01.cir.100.18.1872.

1. Jia H, Abtahian F, Aguirre AD, Lee S, Chia S, Lowe H, et al. In Vivo Diagnosis of Plaque Erosion and Calcified Nodule in Patients With Acute Coronary Syndrome by Intravascular Optical Coherence Tomography. J Am Coll Cardiol. 2013; 62: 1748-1758.

https://doi.org/10.1016/j.jacc.2013.05.071

1. Tearney GJ, Regar E, Akasaka T, Adriaenssens T, Barlis P, Bezerra HG, et al. Consensus Standards for Acquisition, Measurement, and Reporting of Intravascular Optical Coherence Tomography Studies. Journal of the American College of Cardiology. 2012; 59: 1058-72. <https://doi.org/> 10.1016/j.jacc.2011.09.079.
2. Otake H, Shite J, Ikeno F, Shinke T, Teramoto T, Miyoshi N, et al. Evaluation of the peri-strut low intensity area following sirolimus- and paclitaxel-eluting stents implantation: Insights from an optical coherence tomography study in humans. International Journal of Cardiology. 2012; 157: 38-42. <https://doi.org/> 10.1016/j.ijcard.2010.11.006.
3. Araki M, Park S-J, Dauerman HL, Uemura S, Kim J-S, Di Mario C, et al. Optical coherence tomography in coronary atherosclerosis assessment and intervention, Nature Reviews Cardiology. 2022; 19: 684-703. https://doi.org/10.1038/s41569-022-00687-9.
4. Yamamoto MH, Yamashita K, Matsumura M, Fujino A, Ishida M, Ebara S, et al. Serial 3-Vessel Optical Coherence Tomography and Intravascular Ultrasound Analysis of Changing Morphologies Associated With Lesion Progression in Patients With Stable Angina Pectoris. Circ Cardiovasc Imaging. 2017; 10: e006347.

<https://doi.org/> 10.1161/CIRCIMAGING.117.006347.


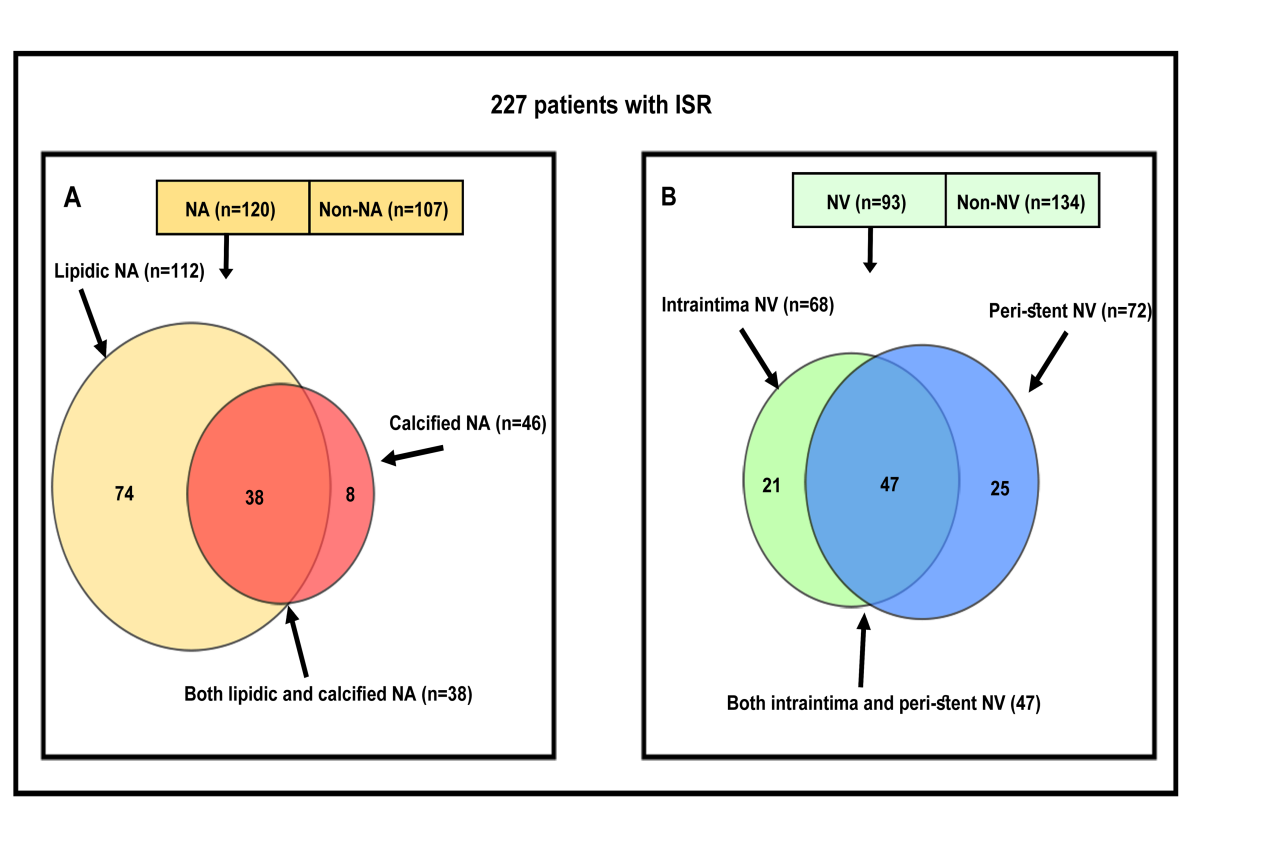
**Supplementary Fig. 1.** **The prevalence of NA and NV.** ISR: in-stent restenosis; NA: neoatherosclerosis; NV: neovascularization.

**Supplementary Table 1. Angiographic characteristics.**

|  | | **Overall**  **(n=227)** | **E-ISR**  **(n=55)** | **L-ISR**  **(n=78)** | **VL-ISR**  **(n=94)** | ***p* value** |
| --- | --- | --- | --- | --- | --- | --- |
|  |  |  |  |  |  |  |
| Length, mm | | 12.10 (8.70–17.90) | 9.50  (8.30–17.60) | 11.85 (8.45–18.23) | 13.40 (9.05–18.00) | 0.195 |
| Reference vessel diameter, mm | | 3.18±0.41 | 3.08±0.35 | 3.21±0.38 | 3.22±0.45 | 0.090 |
| MLD, mm | | 1.14±0.22 | 1.10±0.23 | 1.17±0.20 | 1.13±0.22 | 0.167 |
| Diameter stenosis (%) | | 64.11±5.91 | 64.12±6.89 | 63.31±5.78 | 64.76±5.35 | 0.279 |
| ISR location | |  |  |  |  | 0.914 |
|  | LAD | 143 (63.0) | 34 (61.8) | 50 (64.1) | 59 (62.8) |  |
|  | LCX | 23 (10.1) | 7 (12.7) | 6 (7.7) | 10 (10.6) |  |
|  | RCA | 61 (26.9) | 14 (25.5) | 22 (28.2) | 25 (26.6) |  |
| Restenotic pattern | |  |  |  |  |  |
|  | Focal | 94 (41.4) | 29 (52.7) | 30 (38.5) | 35 (37.2) | 0.145 |
|  | Diffuse | 133 (58.6) | 26 (47.3) | 48 (61.5) | 59 (62.8) | 0.145 |
| Previous stent type | |  |  |  |  | 0.479 |
|  | First-generation DES | 172 (75.8) | 40 (72.7) | 56 (71.8) | 76 (80.9) |  |
|  | New-generation DES | 44 (19.4) | 13 (23.6) | 18 (23.1) | 13 (13.8) |  |
|  | Unknown | 11 (4.8) | 2 (3.6) | 4 (5.1) | 5 (5.3) |  |

Data are expressed as the median [interquartile range], mean±SD (standard deviation), or n (%). DES: Drug eluting stent; E-ISR: early in-stent restenosis; LAD: left anterior descending artery; LCX: left circumflex artery; L-ISR: late in-stent restenosis; MLD: minimal lumen diameter; RCA: right coronary artery; VL-ISR: very late in-stent restenosis.

**Supplementary Table 2. Analysis of the minimum lumen area site by OCT.**

|  | | | **Overall**  **(n=227)** | 1. **ISR**   **(n=55)** | **L-ISR**  **(n=78)** | **VL-ISR**  **(n=94)** | ***p* value** | ***p* value*** | | |
| --- | --- | --- | --- | --- | --- | --- | --- | --- | --- | --- |
|  |  |  |  |  |  |  |  | **①vs.②** | **①vs.③** | **②vs.③** |
| **Quantitative analysis** | | |  |  |  |  |  |  |  |  |
|  | lumen area, mm ^2^ | | 1.64±0.55 | 1.51±0.56 | 1.71±0.53 | 1.66±0.55 | 0.110 |  |  |  |
|  | Stent area, mm ^2^ | | 6.74±1.97 | 6.30±1.88 | 7.12±1.80 | 6.67±2.11 | 0.056 |  |  |  |
|  | Neointimal  area, mm^2^ | | 5.10±1.81 | 4.79±1.75 | 5.41±1.65 | 5.01±1.95 | 0.126 |  |  |  |
|  | Neointimal  burden (%) | | 74.54±8.79 | 74.81±9.91 | 75.34±7.49 | 73.73±9.11 | 0.478 |  |  |  |
| **Qualitative analysis** | | |  |  |  |  |  |  |  |  |
|  | Homogeneous | | 49 (21.6) | 28 (50.9) | 13 (16.7) | 8 (8.5) | <0.001 | <0.001 | <0.001 | 0.104 |
|  | Heterogeneous | | 178 (78.4) | 27 (49.1) | 65 (83.3) | 86 (91.5) | <0.001 | <0.001 | <0.001 | 0.104 |
|  | Layered | | 17 (7.5) | 4 (7.3) | 5 (6.5) | 8 (8.5) | 0.871 |  |  |  |
|  | NA | | 97 (42.7) | 16 (29.1) | 30 (38.5) | 51 (54.3) | 0.007 | 0.263 | 0.003 | 0.039 |
|  |  | Lipidic | 93 (41.0) | 14 (25.5) | 28 (35.9) | 51 (54.3) | 0.001 | 0.202 | 0.001 | 0.016 |
|  |  | Calcified | 16 (7.0) | 2 (3.6) | 5 (6.4) | 9 (9.6) | 0.379 |  |  |  |
|  | TCFA | | 27 (11.9) | 1 (1.8) | 8 (10.3) | 18 (19.1) | 0.006 | 0.080 | 0.002 | 0.105 |
|  | Intimal rupture | | 23 (10.1) | 1 (1.8) | 8 (10.3) | 14 (14.9) | 0.038 | 0.080 | 0.010 | 0.365 |
|  | Plaque erosion | | 16 (7.0) | 2 (3.6) | 5 (6.4) | 9 (9.6) | 0.379 |  |  |  |
|  | Macrophage | | 24 (10.6) | 1 (1.8) | 8 (10.3) | 15 (16.0) | 0.025 | 0.080 | 0.007 | 0.274 |
|  | Cholesterol crystal | | 15 (6.6) | 2 (3.6) | 5 (6.4) | 8 (8.5) | 0.511 |  |  |  |
|  | PLIA | | 21 (12.3) | 4 (7.3) | 9 (11.5) | 8 (8.5) | 0.669 |  |  |  |
|  | NV | | 54 (23.8) | 7 (12.7) | 16 (20.5) | 31 (33.0) | 0.014 | 0.242 | 0.006 | 0.068 |
|  |  | Intraintima | 26 (11.5) | 2 (3.6) | 8 (10.3) | 16 (17.0) | 0.043 | 0.154 | 0.016 | 0.202 |
|  |  | Peri-stent | 30 (13.2) | 5 (9.1) | 9 (11.5) | 16 (17.0) | 0.334 |  |  |  |
|  | Thrombus | | 24 (10.6) | 1 (1.8) | 9 (11.5) | 14 (14.9) | 0.041 | 0.046 | 0.010 | 0.520 |
|  |  | Red | 15 (6.6) | 0 (0.0) | 5 (6.4) | 10 (9.6) | 0.041 | 0.077 | 0.014 | 0.328 |
|  |  | White | 13 (5.7) | 1 (1.8) | 5 (6.4) | 7 (7.4) | 0.389 |  |  |  |

Data are expressed as mean±SD (standard deviation) or n (%). *A *p* value of <0.017 was considered statistically significant. E-ISR: early in-stent restenosis; L-ISR: late in-stent restenosis; NA: neoatherosclerosis; NV: neovascularization; OCT: optical coherence tomography; PLIA: peri-low intensity area; TCFA: thin-cap fibroatheroma. VL-ISR: very late in-stent restenosis. ①: E-ISR; ②: L-ISR; ③: VL-ISR.

**Supplementary Table 3. Patient characteristics between the NA and non-NA [group](javascript:;)s.**

|  | | | | **Overall (n=227)** | **NA (n=120)** | **Non-NA (n=107)** | ***p* value** |
| --- | --- | --- | --- | --- | --- | --- | --- |
|  |  |  |  |  |  |  |  |
| **General information** | | | |  |  |  |  |
|  |  | Age, year | | 64.00 (56.00–71.00) | 64.50 (57.00–71.00) | 62.00 (54.00–70.00) | 0.094 |
|  |  | Male | | 175 (77.1) | 92 (76.7) | 83 (77.6) | 0.872 |
|  |  | Smoking | | 125 (55.1) | 60 (50.0) | 65 (60.7) | 0.104 |
|  |  | Hypertension | | 138 (60.8) | 75 (62.5) | 63 (58.9) | 0.577 |
|  |  | Diabetes mellitus | | 70 (30.8) | 36 (30.0) | 34 (31.8) | 0.772 |
|  |  | LDL-C (mmol/L) | | 2.33 (1.95–2.90) | 2.55 (2.04–3.16) | 2.17 (1.76–2.64) | <0.001 |
|  |  | Creatinine (μmol/L) | | 84.00 (70.00–100.00) | 86.00 (70.25–106.75) | 83.00 (67.00–96.00) | 0.032 |
|  |  | LVEF (%) | | 56.00 (44.00–61.00) | 55.00 (44.00–59.00) | 56.00 (45.00–61.00) | 0.124 |
|  |  | Time from implantation (months) | | 38.00 (13.00–72.00) | 49.00 (24.00–84.00) | 36.00 (11.00–65.00) | 0.017 |
| **CAG finding** | | | |  |  |  |  |
|  | | Length, mm | | 12.10 (8.7–17.90) | 13.20 (8.73–17.88) | 10.70 (8.60–18.00) | 0.178 |
|  |  | Reference vessel  diameter, mm | | 3.18±0.41 | 3.21±0.42 | 3.15±0.39 | 0.315 |
|  |  | MLD, mm | | 1.14±0.22 | 1.12±0.22 | 1.15±0.21 | 0.311 |
|  |  | Diameter stenosis (%) | | 63.48 (60.06–67.68) | 64.34 (60.53–67.99) | 62.60 (59.36–67.12) | 0.069 |
|  |  | Previous stent type | |  |  |  | 0.369 |
|  | |  | First-generation DES | 172 (75.8) | 88 (73.3) | 84 (78.5) |  |
|  |  |  | New-generation DES | 44 (19.4) | 24 (20.0) | 20 (18.7) |  |
|  |  |  | Unknown | 11 (4.8) | 8 (6.7) | 3 (2.8) |  |
| **OCT finding** | | | |  |  |  |  |
|  |  | MLA, mm ^2^ | | 1.64±0.55 | 1.64±0.61 | 1.64±0.48 | 0.928 |
|  |  | Stent area (MLA site), mm ^2^ | | 6.55 (5.36–7.78) | 6.58 (5.63–7.89) | 6.54 (5.16–7.63) | 0.369 |
|  |  | Neointimal area (MLA site), mm^2^ | | 4.80 (3.84–6.12) | 4.88 (4.16–6.16) | 4.75 (3.67–6.05) | 0.253 |
|  |  | Neointimal burden (MLA site), % | | 74.54±8.79 | 75.29±9.15 | 73.71±8.33 | 0.176 |

Data are expressed as the median [interquartile range], mean±SD (standard deviation), or n (%). CAG: coronary angiography; LDL-C: low-density lipoprotein cholesterol; LVEF: left ventricular ejection fractions; MLA: minimum lumen area; MLD: minimal lumen diameter; NA: neoatherosclerosis; OCT: optical coherence tomography.
